# Provisional Association of America

**Published:** 1877-05-20

**Authors:** J. B. Biddle

**Affiliations:** Pres’t


A meeting of the Provisional Association of American Medical Colleges will be held at the Palmer House, Chicago, on Saturday, June 2d, 1877, at 10 o'clock, A. M. All colleges represented at the meeting of the Association held June, 1876, are invited to send delegates to the ensuing meeting, and all chartered medical colleges in the United States recognized as "regular" by the colleges already represented in this Association, are also invited to send delegates from their Faculties to the said meeting.

J. B. BIDDLE, M. D., *Pres't.*

